# 1114. Exploring bactericidal activity and emergence of resistance for carbapenem-resistant *Enterobacterales* (CRE) with and without *Klebsiella pneumoniae* carbapenemase (KPC) treated with optimized meropenem (MEM) and meropenem-vaborbactam (MEV) exposures

**DOI:** 10.1093/ofid/ofad500.087

**Published:** 2023-11-27

**Authors:** Kevin M Squires, Ellen G Kline, Chelsea Jones, Ryan K Shields

**Affiliations:** University of Pittsburgh, Pittsburgh, Pennsylvania; University of Pittsburgh, Pittsburgh, Pennsylvania; UPMC, Pittsburgh, Pennsylvania; University of Pittsburgh, Pittsburgh, Pennsylvania

## Abstract

**Background:**

KPC producing CRE are common in the US; however non-carbapenemase producing (CP) CRE are as common. KPC- and non-CP *Escherichia coli* (ECOL) and *Enterobacter cloacae* complex (ENTCC) demonstrate a range of MEM MICs. The objective of this study was to assess the *in vitro* killing activity of dose-optimized MEM against CRE with or without KPC that demonstrate variable MEM MICs and to determine if vaborbactam potentiates the killing activity of MEM against non-CP CRE.

**Methods:**

8 ECOL and 8 ENTCC were characterized by whole-genome sequencing (**Table**); isolates demonstrated MEM MICs ranging from 1 to 8 mg/L. MEM standard (1g q8h over 30 minutes) and optimized (2g q8h over 3 hours) regimens were tested in 1-compartment and hollow fiber infection models (HFIM); targeted exposures were confirmed by LC-MS/MS. In HFIM, MEV (4g q 8h over 3 hours) was also tested. Resistant subpopulations were selected by agar plates containing MEM at 4x baseline MIC.

**Figure:**
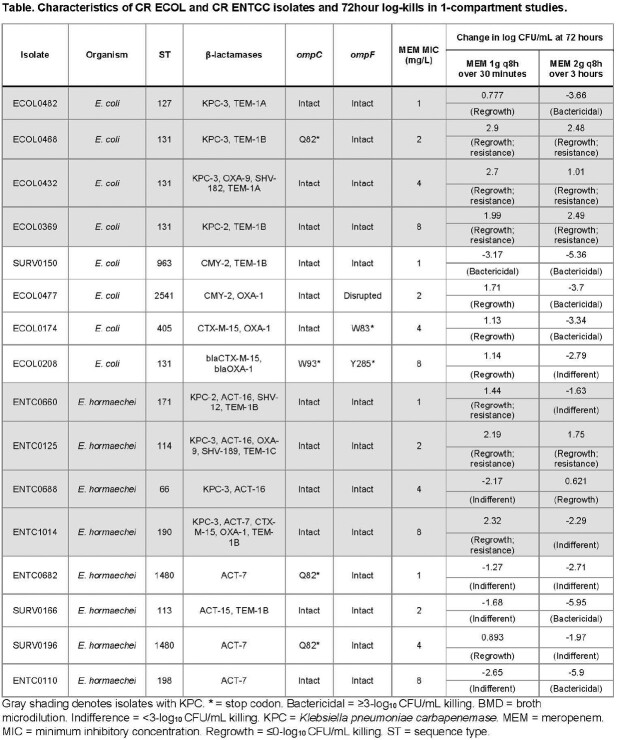

**Results:**

Standard MEM regimens were largely not bactericidal against ECOL (**Figure 1**) or ENTCC (**Figure 2**). Bacterial regrowth occurred more rapidly against KPC compared to non-CP isolates. For MEM optimized regimens, 72-hour log-kills decreased as MEM MICs increased against non-CP ECOL; optimized MEM regimens were not bactericidal against KPC ECOL with MICs ≥2 mg/L. For ENTCC, optimized MEM resulted in greater log-kills against non-CP than KPC isolates at each MIC level. Across all non-CP and KPC isolates with MEM MICs ≤2 mg/L (n=4 each), mean log-kills at 72 hours with optimized MEM were -4.43 and -0.26 logs, respectively (*P*=0.04). Colonies selected on MEM containing agar demonstrated a median (range) MEM MIC of 64 mg/L (16 – >256 mg/L). Next, we compared optimized MEM and MEV in 10 day HFIMs against isolates with MEM MICs of 4mg/L. MEV demonstrated potent bactericidal killing against KPC isolates. Unexpectedly, MEV was also bactericidal against both non-CP isolates, and potentiated MEM killing against ECOL0174 (**Figure 3**).

**Figure 1.**
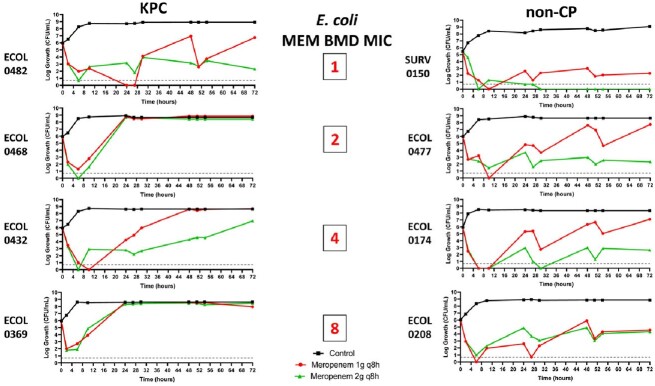
Log-growth of clinical CR ECOL exposed to humanized meropenem exposures during 72 hour 1-compartment study.

Standard dosing MEM 1g q8h, 30 minute infusion (red). Optimized dosing MEM 2g q8h, 3 hour infusion (green). Limit of detection is 0.7 log colony forming units (CFU)/mL, denoted by dashed line. BMD = broth microdilution. KPC = Klebsiella pneumoniae carbapenemase.

**Figure 2.**
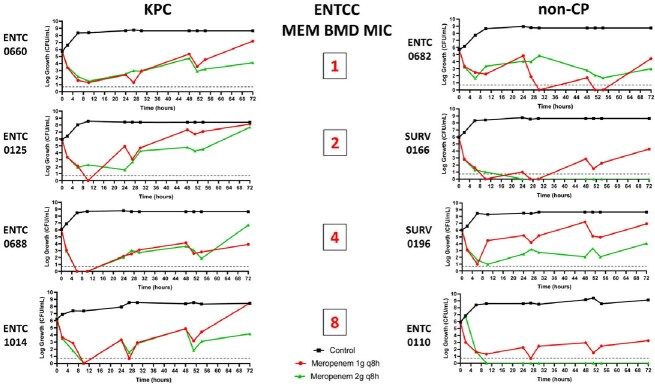
Log-growth of clinical CR ENTCC exposed to humanized meropenem exposures during 72 hour 1-compartment study.

Standard dosing MEM 1g q8h, 30 minute infusion (red). Optimized dosing MEM 2g q8h, 3 hour infusion (green). Lower limit of detection is 0.7 log CFU/mL, denoted by dashed line. BMD = broth microdilution. KPC = Klebsiella pneumoniae carbapenemase

**Figure 3.**
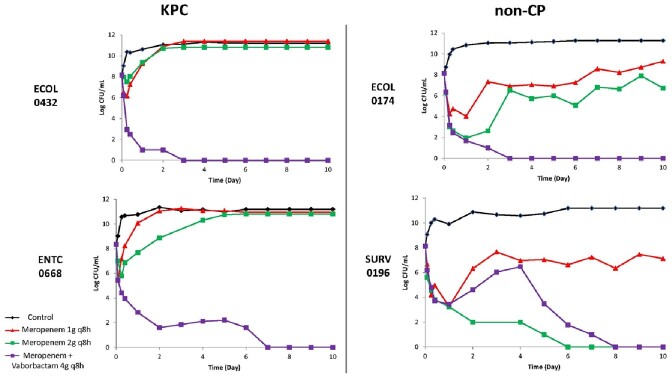
Log-growth of clinical CR ECOL and ENTCC (meropenem MIC=4mg/L) exposed to humanized exposures of meropenem and meropenem-vaborbactam in 10 day hollow fiber infection model.

Standard dosing meropenem 1g q8h, 30 minute infusion (red). Optimized dosing meropenem 2g q8h, 3 hour infusion (green), or meropenem-vaborbactam 4g q8h, 3 hour infusion (purple). Lower limit of detection is 0.7 log CFU/mL. MIC = minimum inhibitory concentration.

**Conclusion:**

Our data show *in vitro* killing of CR ECOL and ENTCC is dependent on MEM MICs and presence of KPC. Even at low MICs, optimized MEM exposures were ineffective against KPC isolates. Against non-CP CRE, killing decreases as a function of increased MEM MIC. MEV may play an additive role in killing against non-CP isolates that are not killed by MEM alone.

**Disclosures:**

**Ryan K. Shields, PharmD, MS**, Allergan: Advisor/Consultant|Cidara: Advisor/Consultant|Entasis: Advisor/Consultant|GSK: Advisor/Consultant|Melinta: Advisor/Consultant|Melinta: Grant/Research Support|Menarini: Advisor/Consultant|Merck: Advisor/Consultant|Merck: Grant/Research Support|Pfizer: Advisor/Consultant|Roche: Grant/Research Support|Shionogi: Advisor/Consultant|Shionogi: Grant/Research Support|Utility: Advisor/Consultant|Venatorx: Advisor/Consultant|Venatorx: Grant/Research Support

